# YouTube Videos Demonstrating the Nasopharyngeal Swab Technique for SARS-CoV-2 Specimen Collection: Content Analysis

**DOI:** 10.2196/24220

**Published:** 2021-01-14

**Authors:** Kyohei Itamura, Arthur Wu, Elisa Illing, Jonathan Ting, Thomas Higgins

**Affiliations:** 1 Division of Otolaryngology - Head and Neck Surgery Cedars-Sinai Medical Center Los Angeles, CA United States; 2 Department of Otolaryngology - Head and Neck Surgery Indiana University Indianapolis, IN United States; 3 Department of Otolaryngology - Head and Neck Surgery and Communicative Disorders University of Louisville Louisville, KY United States; 4 Rhinology, Sinus & Skull Base Kentuckiana Ear, Nose, and Throat Louisville, KY United States

**Keywords:** COVID-19, coronavirus, SARS-coV-2, nasopharyngeal swab, viral testing, PCR, YouTube, infodemiology, digital epidemiology, testing, diagnostic, content analysis, video, error

## Abstract

**Background:**

Real-time polymerase chain reaction using nasopharyngeal swabs is currently the most widely used diagnostic test for SARS-CoV-2 detection. However, false negatives and the sensitivity of this mode of testing have posed challenges in the accurate estimation of the prevalence of SARS-CoV-2 infection rates.

**Objective:**

The purpose of this study was to evaluate whether technical and, therefore, correctable errors were being made with regard to nasopharyngeal swab procedures.

**Methods:**

We searched a web-based video database (YouTube) for videos demonstrating SARS-CoV-2 nasopharyngeal swab tests, posted from January 1 to May 15, 2020. Videos were rated by 3 blinded rhinologists for accuracy of swab angle and depth. The overall score for swab angle and swab depth for each nasopharyngeal swab demonstration video was determined based on the majority score with agreement between at least 2 of the 3 reviewers. We then comparatively evaluated video data collected from YouTube videos demonstrating the correct nasopharyngeal swab technique with data from videos demonstrating an incorrect nasopharyngeal swab technique. Multiple linear regression analysis with statistical significance set at *P*=.05 was performed to determine video data variables associated with the correct nasopharyngeal swab technique.

**Results:**

In all, 126 videos met the study inclusion and exclusion criteria. Of these, 52.3% (66/126) of all videos demonstrated the correct swab angle, and 46% (58/126) of the videos demonstrated an appropriate swab depth. Moreover, 45.2% (57/126) of the videos demonstrated both correct nasopharyngeal swab angle and appropriate depth, whereas 46.8% (59/126) of the videos demonstrated both incorrect nasopharyngeal swab angle and inappropriate depth. Videos with correct nasopharyngeal swab technique were associated with the swab operators identifying themselves as a medical professional or as an Ear, Nose, Throat–related medical professional. We also found an association between correct nasopharyngeal swab techniques and recency of video publication date (relative to May 15, 2020).

**Conclusions:**

Our findings show that over half of the videos documenting the nasopharyngeal swab test showed an incorrect technique, which could elevate false-negative test rates. Therefore, greater attention needs to be provided toward educating frontline health care workers who routinely perform nasopharyngeal swab procedures.

## Introduction

Qualitative real-time polymerase chain reaction (RT-PCR) of nasopharyngeal secretions is the gold standard for testing respiratory viruses, including SARS-CoV-2 [1]. However, major concerns have been raised regarding false-negative rates of RT-PCR tests in community testing locations [2]. An early retrospective review of community hospital testing performed in China reported a test sensitivity of only 71% [3]. Although the false-negative results could be attributed to various reasons, including laboratory errors, patient misidentification, and inadequate collection of specimens, improper technique resulting in the swabs not reaching the target site of the nasopharynx is a potentially pervasive but modifiable error.

The trajectory from the nostril to the nasopharynx is often presumed to be along the dorsum of the nose, likely because of the visual appearance of the external nose. In reality, the correct trajectory is along the floor of the nose in the direction back toward the ear. Deviating from this trajectory can lead to pain from contacting a deviated septum or nasal turbinate or failure to reach the nasopharynx. Although the Center for Disease Control and Prevention has provided guidance regarding the proper nasopharyngeal swab (NPS) technique, vivid descriptions of painful patient experiences are currently commonplace in the media [4].

Although many health centers around the world are likely providing proper training to frontline health care workers, there is concern that improper NPS techniques for specimen collection may lead to false-negative results in RT-PCR tests [5]. This is a significant concern, as false-negative test results underestimate the prevalence of COVID-19 and give a false sense of security to patients as well as the health care workers caring for them [6]. Moreover, the use of improper NPS techniques limits public health efforts in identifying and contact tracing the spread of the virus. Thus, with the widely established use of NPS as a large-scale screening tool for COVID-19 and other respiratory viral diseases, ensuring a proper collection technique is used is essential in yielding sensitive test results [7],

Accordingly, the purpose of this study was to determine how the NPS technique for SARS-CoV-2 is instructed or demonstrated and how the NPS test is administered in real life by reviewing videos hosted on the web-based video-sharing platform YouTube (Google, LLC) [8].

## Methods

### Sample Size Determination

The sample size calculation was performed using the Kelsey methodology for cross-sectional study with a power of 80% and 2-sided confidence level of 0.05%; the exposure ratio was estimated to be 1:1 and odds ratio was estimated to be 3. The total estimated sample size was 64.

### YouTube Database Search

YouTube is a widely used social media database of videos uploaded by the general public. Due to the broad accessibility of this database, there was no requirement for research approval by the Institutional Review Board–Human Subjects. The terms “nasopharyngeal swab,” “nasopharyngeal test,” “nasal swab,” “coronavirus swab,” “coronavirus test,”“covid swab,” and “covid test” were used to query the YouTube video database [8]. The query was filtered by setting the “sort by” filter to “by upload date” to compile all videos published from January 1, 2020, to May 15, 2020. Inclusion and exclusion criteria were defined to screen all search results. The inclusion criterion was that the NPS test is performed on screen with visualization of swab insertion into either naris. The exclusion criteria were duplicate videos, non–COVID-19 swab indication, and swab testing intended for anatomical regions other than the nasopharynx (eg, anterior nasal swabbing).

### Video Evaluation and Data Collection

Three faculty rhinologists individually reviewed the selected NPS demonstration videos for swab angle and swab depth. Swab angle was assessed as either “along the nasal floor” or “not along the nasal floor.” Swab depth was assessed as either “appropriate depth” or “inappropriate depth.” All reviewers were blinded to each other’s assessments. The following ancillary video data were collected: video author type (“medical,” including physician, registered nurse, physician’s assistant, or nurse practitioner, vs “nonmedical”), operator type (“medical” vs “nonmedical”), type of video (“instructional vs “noninstructional”), specialty (“otolaryngology” vs “other”), country of origin (“United States [USA]” vs “other”), number of likes, number of author subscribers, time in nasal cavity, time at nasopharynx, and video post date relative to May 15, 2020.

### Statistical Analysis

Interrater reliability among the 3 reviewers was assessed using Fleiss’ Kappa. The overall score for swab angle and swab depth for each NPS demonstration video was determined based on the majority score with agreement between at least 2 of the 3 reviewers. Video data were also compared between YouTube videos demonstrating the correct NPS technique and those demonstrating an incorrect NPS technique. Multiple linear regression analysis was performed to determine predictive variables among video data for videos demonstrating the correct NPS technique. Statistical significance was set at *P*=.05. All statistical analyses were performed on Microsoft Excel (Microsoft Corp.).

## Results

**Figure 1:**
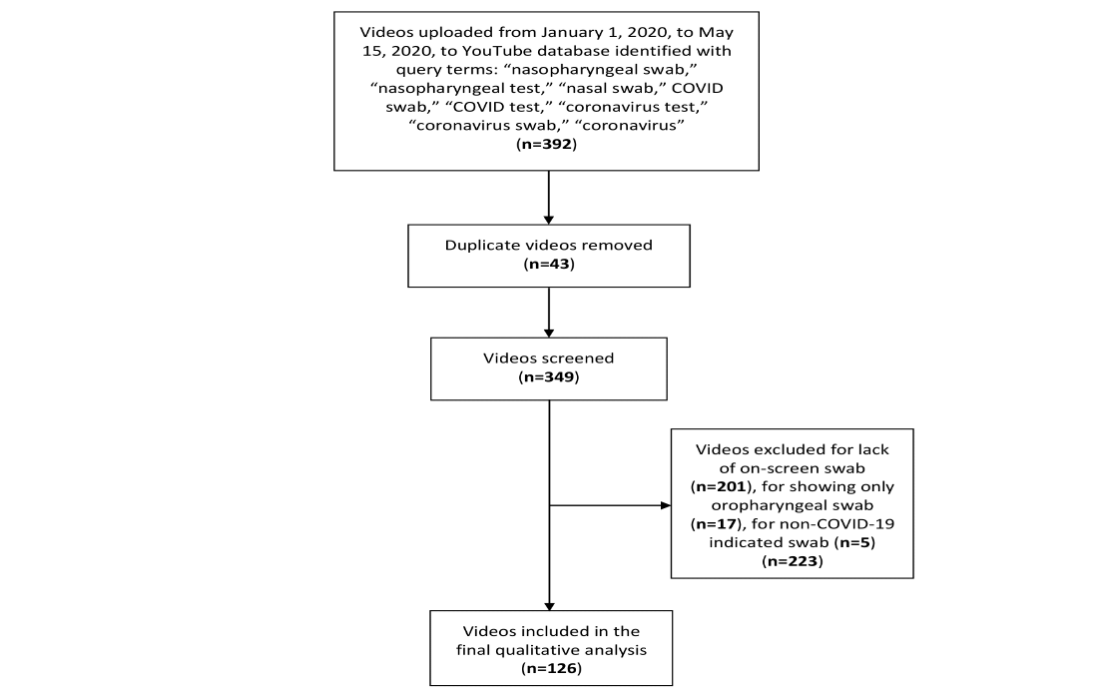
PRISMA (Preferred Reporting Items for Systematic Reviews and Meta-analyses) flow diagram.

The final qualitative analysis included 126 independent, unique videos. The video selection process, including screening for inclusion and exclusion criteria, is summarized in Figure 1.

The κ value indicating interrater reliability for the 3 reviewers was 0.66 for swab angle (*P*<.001; 95% CI=0.56-0.76) and 0.68 for swab depth (*P*<.001; 95% CI=0.58-0.78). For the assessment of swab angle, there was complete agreement among all reviewers, with all 3 scores consistent for 74.6% (94/126) of all videos. For the assessment of swab depth, there was complete agreement among all 3 reviewers for 76.1% (96/126) of all videos.

**Figure 2:**
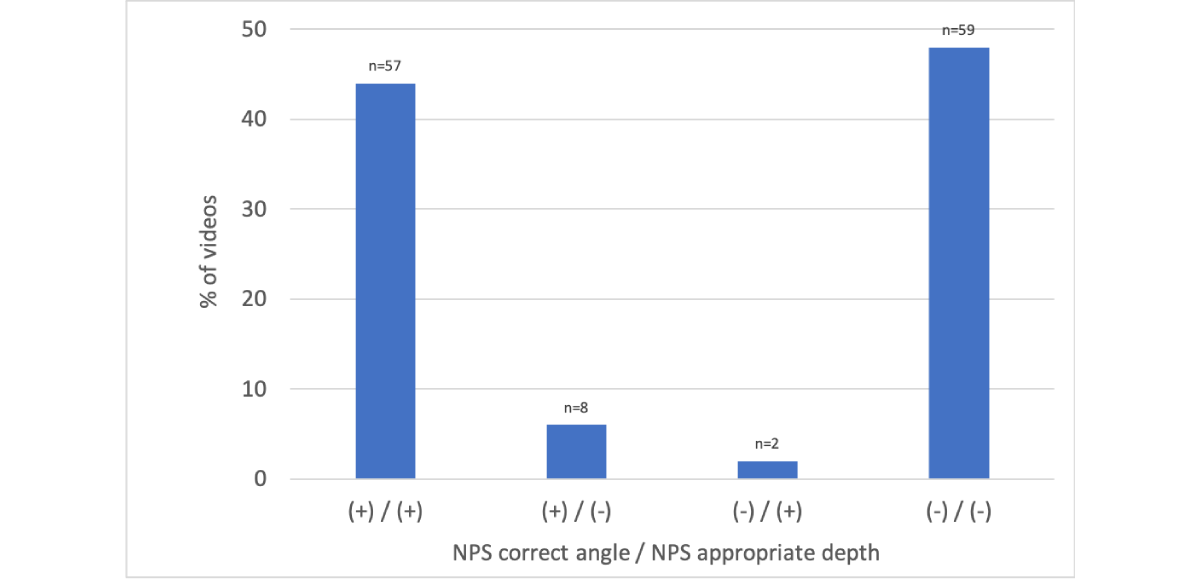
Percentage of YouTube videos demonstrating correct or incorrect nasopharyngeal swab angle and/or appropriate or inappropriate nasopharyngeal swab depth. NPS: nasopharyngeal swab.

Moreover, we found that 52.3% (66/126) of all NPS demonstration videos had the correct angle, and 46% (58/126) showed appropriate depth (Figure 2). In addition, 45.2% (57/126) of all videos had both correct NPS angle and appropriate depth, whereas 46.8% (59/126) of the videos had both incorrect NPS angle and inappropriate depth. We observed concordance between the swab angle and depth (ie, correct swab angle with appropriate swab depth or incorrect swab angle with inappropriate swab depth) in 92% (116/126) of the videos. The agreement between these measures was nearly equivalent with regard to both measures being correct compared with both measures being incorrect. In the remaining approximately 8% (10/126) of the videos, 8 videos demonstrated correct swab angle but inappropriate swab depth, and the remaining 2 videos demonstrated incorrect swab angle but appropriate swab depth.

Table 1 compares the video data between videos demonstrating NPS with correct technique (both correct swab angle and appropriate swab depth) and those demonstrating NPS with incorrect technique (incorrect swab angle and/or inappropriate swab depth). We found that approximately half of the videos with the correct NPS technique were from a medical video author, instructional in nature, and/or of US origin. Videos demonstrating the correct NPS technique also were posted onto YouTube closer to May 15, 2020, compared to those demonstrating an incorrect technique. ENT-related providers were only found in videos demonstrating the correct NPS technique. Video viewership metrics, including number of views, likes, and author subscriber count, varied widely between videos demonstrating correct and incorrect NPS techniques. For correctly performed NPS techniques, the median time at the nasopharynx was 4 seconds. By definition, no time was spent at the nasopharynx in incorrectly performed NPS techniques, with regard to both incorrect depth and/or angle.

**Table 1:** Comparison of video data of YouTube videos demonstrating correct nasopharyngeal swab technique with those demonstrating incorrect nasopharyngeal swab technique

Data type			Correct NPS^a^ technique (n=57)			Incorrect NPS technique (n=69)		
**Video author type, n (%)**								
	Medical (academic/nonacademic physician, nonphysician health care worker, medical group/entity)			29 (51)			27 (39)	
	Nonmedical (media, layperson)			28 (49)			42 (61)	
**Swab operator type, n (%)**								
	Medical (explicit identification as MD, physician’s assistant, nurse practitioner, registered nurse)			55 (96)			59 (86)	
	Unidentified			2 (4)			10 (14)	
**Video type, n (%)**								
	Instructional (for teaching purposes, demonstrational)			32 (56)			33 (48)	
	Real-world test			25 (44)			36 (52)	
**Swab operator specialty, n (%)**								
	ENT (ear, nose, throat)			9 (16)			0 (0)	
	Non-ENT (emergency medicine, primary care, unidentified)			48 (84)			69 (100)	
**Swab collection location, n (%)**								
	Drive-through			15 (26)			26 (38)	
	Non drive-through (clinic, urgent/emergency care, walk-in testing site)			42 (74)			43 (62)	
**Video country of origin, n (%)**								
	USA			29 (51)			41 (60)	
	Rest of the world			28 (49)			28 (40)	
Time for which the swab was inserted in the nose (seconds), median (IQR)		11 (7, 16)			8 (6, 13)			
Number of views, median (IQR)		111.5 (883, 3502)			863 (185, 36618)			
Number of likes, median (IQR)		11 (0, 38.5)			9 (1.5, 106)			
Percentage of likes over total views, median (IQR)		0.6 (0, 1.7)			0.5 (0.2, 1.3)			
Number of channel subscribers, median (IQR)		309 (19.5, 2285)			1690 (24.5, 20,700)			
Video post date (number of days before May 15, 2020), median (IQR)		22 (7, 38)			32 (16, 42)			
Time at nasopharynx (seconds), median (IQR)			4 (1.5, 7)			N/A^b^		

^a^NPS: nasopharyngeal swab.

^b^N/A: not applicable.

Multiple linear regression analysis was performed on video data for videos demonstrating the correct NPS technique as the reference dependent variable (Table 2). The correct NPS technique was associated with the NPS operator identifying as a medical professional and, additionally, as a provider within the ENT specialty. There was also a significant association between correct NPS technique and recency of video post date relative to May 15, 2020.

**Table 2:** Multiple linear regression analysis of video data (reference dependent variable: correct nasopharyngeal technique). Italicized values indicate statistical significance.

Data type	Standardized β coefficient	95% confidence interval	*P* value
Author type (ref: medical)	−.079	−0.34 to 0.19	.55
Swab operator (ref: medical)	.23	0.040-0.41	*.018*
Video type (ref: instructional)	.046	−0.24 to 0.34	.75
Specialty (ref: ENT)	.28	0.11-0.46	*.002*
Testing location (ref: drive-through)	−.094	−0.30 to 0.11	.37
Country (ref: USA)	−.032	−0.22 to 0.15	.74
Longer time in nose	−.0074	−0.26 to 0.11	.42
Number of video views	.026	−0.58 to 0.63	.93
Number of video likes	−.072	−0.68 to 0.53	.82
Ratio of likes over views	.024	−0.15 to 0.20	.80
Number of video channel subscribers	.16	−0.011 to 0.33	.07
Video post date (Number of days before May 15, 2020)	.27	0.076-0.46	*.007*

## Discussion

### Principal Findings

This study demonstrates that almost half (46.8%) of all NPS demonstration videos reviewed showed incorrect swab angle and inappropriate depth, as judged by 3 rhinologists. The noninstructional videos provide a broad vantage point of how testing is actually performed in the real world, so it is alarming to find that the proper technique may be so infrequently used. Moreover, 51% of all instructional videos demonstrated an improper technique. Although it is unknown how many of these instructional videos are actually being used by viewers for the purposes of learning the NPS technique, it still highlights the fact that those claiming to be experts, whether local, national or otherwise, may not have a complete understanding of the NPS technique. Furthermore, there were no statistically significant differences between the viewership of videos demonstrating the correct technique and those demonstrating incorrect NPS techniques. This finding is consistent with previously published work, especially in the early months of the COVID-19 pandemic showing the pervasiveness of uncredentialed, low-quality media publicly available on the internet [9]. These results are not totally unexpected given the lack of sinonasal anatomic knowledge most NPS operators have and the inherent difficulty of navigating the septum and turbinates to reach the nasopharynx. It is noteworthy that all videos of otolaryngologists performing or instructing the NPS were done correctly. These findings emphasize the onus of the otolaryngology field to educate our colleagues on sinonasal anatomy and proper NPS technique during the ongoing COVID-19 pandemic.

Although lower respiratory samples such as bronchoalveolar lavage and sputum samples have higher viral loads in patients with SARS-CoV-2, NPS testing is considered the best alternative over other minimally invasive specimen collection options such as oropharyngeal swabs, blood samples, or stool samples [1]. NPS is widely used to test for other respiratory viral infections and has supplanted nasopharyngeal aspirates for its accuracy and convenience in this setting [10]. However, poor techniques used for the NPS method may convert this test into a simple nasal swab. The NPS test is inherently uncomfortable for the patient even with good technique, and patients or the NPS operator may retract prematurely before the swab reaches the correct location and saturates with mucus. There has been limited attention paid towards the impact proper technique has on the accuracy of results in NPS testing even with regard to influenza or other respiratory viral testing.

Although a comparison of viral loads between the nasal cavity and the nasopharynx has not been reported for SARS-CoV-2, significant differences in viral loads have been demonstrated for other viruses; nevertheless, this is not direct evidence that NPS technique may affect testing accuracy [11,12]. However, NPS sampling technique has been shown to potentially affect false-negative rates in SARS-CoV-2 in a single study [13]. Ma et al [14] demonstrated significantly improved test accuracy and patient comfort when patients were tested in a supine position compared to in a sitting position. Of note, an increasing number of tests are performed via drive-through testing to provide convenience, increase throughput, and adhere to social distancing recommendations [14]. Despite the rapid adoption of this modality, there has not been substantial review of its effect on testing accuracy, and patient and operator positioning may not be optimized for proper NPS technique.

Because of these multiple unknowns in the current SARS-CoV-2 testing climate, we feel that specimen collection technique is at least one aspect that could be easily remedied. Three points need to be emphasized to frontline health care workers performing the NPS technique: trajectory angle, depth, and patient expectations. The swab should be angled to follow the floor of the nose, and the depth required to reach the nasopharynx, approximately 9-10 cm in adults, is often surprising to non-otolaryngologists [7]. In many cases, this means that almost the entire length of the swab is inserted into the nasal cavity with only a small portion left to hold outside the nose. Finally, both the patient and the operator should set proper expectations for the procedure: the NPS is uncomfortable but should not cause sharp or severe pain. Such discomfort should indicate to the operator that anatomic obstruction such as a deviated septum is occluding the pathway, and a modified trajectory or contralateral approach should then be attempted.

### Study Limitations

This study has some limitations, including the lack of consistent video angle and quality. The types of videos ranged from professionally produced instructional videos to “selfies” posted by patients themselves. Although this inconsistency could potentially introduce difficulty in the judging NPS technique being demonstrated, we excluded videos that were clearly difficult to be analyzed. Nevertheless, although a moderate level of interrater agreement was demonstrated by Fleiss’ Kappa analysis, the remaining discordance may be attributable to the large variety in video quality. We believe that the inclusion of a wide array of video types would allow for a more complete view of real-life NPS testing across the globe. Additionally, there was no way to correlate proper or improper NPS technique with false-positive or false-negative testing rates. Finally, reproducibility of the results may be limited due to the involvement of only 3 reviewers, although all of them were board-certified otolaryngologists with fellowship training in rhinology.

### Conclusions

The majority of NPS demonstration videos evaluated in this study used an improper technique. This technical deficiency may affect false-negative rates for SARS-CoV-2 testing. Therefore, based on these findings, we suggest that otolaryngologists should work to educate their medical colleagues and frontline health care workers who perform NPS techniques about relevant anatomy and technical considerations.

## Abbreviations

ENT: ear, nose, throat
NPS: nasopharyngeal swab
PRISMA: Preferred Reporting Items for Systematic Reviews and Meta-analysis
RT-PCR: reverse transcription–polymerase chain reaction
